# Defining a CMV viral load threshold for pre‐emptive therapy in paediatric haematopoietic stem cell transplant recipients

**DOI:** 10.1111/bjh.20254

**Published:** 2025-07-09

**Authors:** Amedine Duret, Oscar Charles, Ben K. Margetts, John Booth, Julianne R. Brown, Timothy Best, Sneha Fernandes, Persis Amrolia, Robert Chiesa, Juliana Silva, Austen Worth, Joseph F. Standing, Kanchan Rao, Elizabeth Whittaker, Eliza Gil, Judith Breuer

**Affiliations:** ^1^ Department of Paediatric Infectious Diseases Imperial College London London UK; ^2^ Infection, Immunity, and Inflammation, Institute of Child Health University College London London UK; ^3^ Centre for Computation, Mathematics, & Physics in the Life Sciences and Experimental Biology University College London London UK; ^4^ Digital Research Environment Great Ormond Street Hospital for Children NHS Foundation Trust London UK; ^5^ Microbiology, Virology and Infection Prevention and Control Great Ormond Street Hospital for Children NHS Foundation Trust London UK; ^6^ Department of Blood and Marrow Transplantation Great Ormond Street Hospital for Children NHS Foundation Trust London UK; ^7^ Pharmacy Department Great Ormond Street Hospital for Children NHS Foundation Trust London UK; ^8^ Department of Academic Paediatrics Imperial College London UK; ^9^ Division of Infection and Immunity University College London London UK

**Keywords:** BMT paed, cytomegalovirus, HSC transplantation, virology

## Abstract

Cytomegalovirus (CMV) infection is a significant complication in paediatric haematopoietic stem cell transplant (HSCT), with substantial morbidity and mortality. While pre‐emptive treatment guided by CMV viral load thresholds helps prevent end‐organ disease, paediatric protocols have largely been extrapolated from adult populations without robust validation. This prospective interventional study aimed to determine an optimal viral load threshold for initiating pre‐emptive CMV therapy in paediatric HSCT patients and test the impact of this threshold in real‐world practice. Initially, 219 paediatric HSCT patients from 2009 to 2016 were considered, with weekly CMV quantitative polymerase chain reaction (qPCR) monitoring and treatment initiation at 2500 IU/mL. A mathematical model was developed to analyse CMV kinetics, morbidity and mortality, suggesting a threshold of 1000 IU/mL would be most suitable to prevent adverse outcomes. Subsequently, the new treatment threshold was implemented, and outcomes were then compared with a second cohort of 344 patients treated under the new guidelines from 2017 to 2021. There were significant reductions in CMV viral loads, CMV‐associated end‐organ disease (27/117, 23.1% vs. 4/98, 4.1%) and mortality (36/117, 30.8% vs. 19/98, 19.4%). These findings support a new evidence‐based viral load threshold of 1000 IU/mL or less for pre‐emptive treatment in paediatric HSCT recipients to optimise clinical outcomes.

## Graphical abstract

Cytomegalovirus (CMV) infection and disease are frequent complications following haematopoietic stem cell transplantation (HSCT) in children. A common prevention strategy involves regular monitoring of CMV viral load and initiating antiviral therapy once a predefined threshold is exceeded. In this study, survival analysis of a paediatric HSCT cohort identified an optimal threshold of 1000 IU/mL to minimise mortality. Implementation of this threshold in clinical practice was associated with reduced CMV‐related morbidity and mortality, as well as an overall decrease in HSCT‐related mortality at the study centre.

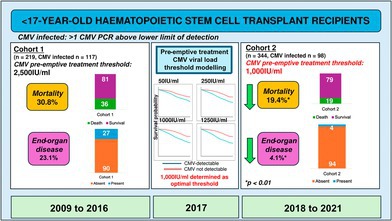

## INTRODUCTION

Cytomegalovirus (CMV) infection and disease are frequent complications of haematopoietic stem cell transplants (HSCT) in paediatric patients, with an incidence of up to 60% for infection and 5%–7% for end‐organ disease.^1^, ^2^, ^3^ The two strategies to prevent CMV disease are prophylaxis and pre‐emptive treatment. Prophylaxis has been suggested to delay recovery of CMV‐specific T‐cell immunity, leading to an increase in late CMV disease.^4^ Furthermore, a randomised controlled trial found no benefit to valganciclovir prophylaxis compared to pre‐emptive therapy strategies,^5^ and trials of either aciclovir or famciclovir prophylaxis in paediatric HSCT patients did not show a reduction in the risk of CMV viraemia.^6^ Letermovir prophylaxis has a good efficacy and safety profile in adults; however, it is not licensed in children under 12.^7^

Pre‐emptive treatment has been the mainstay of CMV disease prevention in paediatric HSCT patients. Blood CMV markers (pp65 antigen assays or CMV quantitative polymerase chain reaction (qPCR)) are regularly monitored, and treatment is initiated if a pre‐stated threshold is crossed. As the available pharmacological treatment for CMV has significant side effects, it is judicious to have a treatment strategy that avoids overtreatment at low CMV viraemia levels where the risk of CMV disease is low, while ensuring that patients at risk of progressing towards end‐organ disease receive early therapy.

CMV viral load measured by qPCR is accepted as a valid surrogate marker for CMV disease in clinical trials in transplant patients.^6^ While inter‐laboratory variability in CMV quantification by qPCR makes cross‐institution comparisons challenging,^8^ this has largely been addressed through accepted international standards.^9^ CMV viral load in the initial phase of infection and the rate of viral load increase are correlated with CMV disease in adult transplant patients.^10^ However, the relationship between viral load and CMV disease is non‐linear, as little end‐organ disease is seen until critical viraemia is reached,^11^, ^12^ with 10 000 copies/mL (c/mL) recognised as the CMV level at which end‐organ disease occurs.^11^ A randomised controlled trial in adult solid and stem cell transplant patients found that 3000 c/mL was an appropriate threshold to initiate pre‐emptive therapy to prevent the threshold for end‐organ disease being reached and minimise morbidity and mortality.^13^

There is a lack of equivalent evidence to underpin the selection of appropriate thresholds for pre‐emptive CMV treatment in paediatric transplant recipients. An Australasian survey of practice revealed that a wide range of qPCR thresholds were used in paediatric HSCT patients, with five of six centres starting treatment after one CMV PCR >1000 c/mL and the remaining centre after one CMV PCR >10000 c/mL.^14^ A similar survey of European bone marrow transplant centres highlighted similar disparities, with 33/44 centres using 1000 c/mL as a cut‐off, 4/44 centres using 5000 c/mL and the rest not disclosing their thresholds.^15^

We report the novel application of mathematical modelling approaches to inform the treatment threshold for pre‐emptive CMV therapy in paediatric HSCT patients. Mathematical modelling of the CMV kinetics in adult HSCT patients with untreated CMV infection has been performed to understand host immune response to CMV viraemia,^16^ but these approaches have not previously been used to inform therapeutic approaches in either adults or children.

We first undertook a retrospective cohort study of CMV viraemia in relation to morbidity and mortality in a cohort of 219 paediatric HSCT patients at a bone marrow centre in the United Kingdom. CMV viral loads were tested weekly by qPCR until the CD4+ counts recovered to >300 cells/μL. Antiviral treatment was started following two consecutive CMV levels of 10 000 c/mL (equivalent to 2500 IU/mL) or above in line with recommendations published in a study conducted in paediatric HSCTs.^17^ A mathematical model was derived incorporating data from this cohort on CMV viral load, morbidity and mortality, and this was used to estimate an optimal threshold at which CMV treatment should be initiated to minimise adverse outcomes. Based on the results, hospital policy was changed in 2017 to recommend pre‐emptive CMV treatment at the optimal threshold suggested by the model. All other aspects of the policy were unchanged. Morbidity and mortality data collected from 344 HSCT recipients at the same UK centre from 2017 to 2021, treated according to the new threshold guidelines, were used to audit the impact of the changes and, in doing so, validate the model assumptions.

## METHODS

To characterise the role of CMV infection and disease on morbidity and mortality in children undergoing HSCT, we undertook a retrospective review of all patients aged 17 and below, receiving HSCTs at Great Ormond Street Hospital London from 1 December 2009 to 31 December 2016 (*n* = 219). These patients make up ‘Cohort 1’. Viral dynamics and their association with mortality and CMV disease were analysed and modelled as described below to derive a treatment threshold at which to initiate antiviral treatment for CMV.

A new treatment threshold, based on the mathematical modelling performed on Cohort 1, was brought into clinical practice on 1 January 2017. Following the introduction of the treatment threshold, virological and clinical data were collected for all patients aged 17 and below receiving HSCTs from 1 January 2017 to 14 May 2021 (*n* = 344), termed ‘Cohort 2’.

### Virological monitoring

CMV viral loads in whole blood were tested as part of routine clinical care by a laboratory‐developed (LDT) qPCR assay as previously described,^17^ on a weekly basis until circulating CD4+ counts recover to >300 cells/μL. The qPCR assay was calibrated to the first international WHO standard, reporting viral load in both c/mL and IU/mL for clinical care with a lower limit of quantification (LLOQ) of 50 IU/mL (equivalent to 200 c/mL). If CMV was detected, testing was increased to a minimum of two samples/week. For Cohort 1, viral loads of >10000 c/mL (2500 IU) were confirmed by a second test before starting treatment. Three patient datasets were generated from Cohort 1, a ‘transplant’ dataset (*n* = 219), a ‘CMV infected’ dataset (*n* = 117) and a ‘CMV treated’ dataset (*n* = 68). For Cohort 2, a ‘transplant’ dataset (*n* = 344), ‘CMV‐infected’ dataset (*n* = 98) and ‘CMV‐treated’ dataset (*n* = 64) were created. Viral area under the curve (AUC) is an expression of the cumulative viral load over the entire transplant period for each patient.

### Time taken to exhibit CMV infection post‐HSCT in children

To determine the CMV burden post‐HSCT and to quantify the time taken to reach specific viral load values, the time between transplant and the first CMV observation above set cut‐off values was calculated as a cumulative distribution function for all 219 patients in the ‘transplant’ dataset of Cohort 1. Cut‐off values reported were selected to highlight the first observation at or above the CMV LLOQ, as well as five higher viral load values selected to demonstrate viral disease progression above the LLOQ.

### Quantifying the presence of disease in relation to CMV infection

Clinical records were used to determine mortality statistics in all patients in Cohort 1 and Cohort 2. CMV‐associated disease (encephalitis, pneumonitis, retinitis, gastro‐intestinal disease) was identified and classified according to the evidence definitions set out by the Disease Definitions Working Group of the Cytomegalovirus Drug Development Forum.^10^

### Determining optimal thresholds for pre‐emptive treatment

We used a time‐to‐event (Kaplan–Meier) model to elucidate any potential relationship between the covariates of peak viral load and mortality. Survival data for all 219 patients in Cohort 1 were also modelled by fitting a Cox proportional hazards function to dates of death post‐transplant for both CMV positive and negative patients. Since Kaplan–Meier may suffer from bias with this type of data, a Mantel–Byar analysis, which addresses potential immortal time bias, was also performed to validate the findings. This test allows for group membership of an individual to change from CMV negative to CMV positive during follow‐up, which occurred upon exceeding cut‐off values.^18^, ^19^

The presence of one or more CMV peak viral loads above or equal to the putative treatment threshold between 50 IU and 5000 IU/mL was used as a separator, assigning patients to one of two groups: (1) those who remained CMV negative, or whose peak viral load fell below the threshold (forming the data for the null model), (2) those who exhibited a CMV viral load above or equal to the threshold (forming the data for the alternative model).

Following assignment, separate hazard functions were fitted to the two groups and a likelihood ratio test was used to quantify the likelihood that the null model would fit the data from the alternative model. Cut‐off values used in this analysis began at the CMV LLOQ and were followed by a selection of values used to demonstrate survival at a range of thresholds.

### Statistical analyses

All data cleaning, transformations and analyses were conducted in the statistical computing environment, R (version 4.1.2), using the trapz package for computing AUCs, the survival package for fitting hazard functions, RcmdrPlugin.EZR for the Mantel–Byar test^19^ and the ggplot2 package for visualisations. As the viral load data were non‐normally distributed when running pairwise comparisons, a Wilcoxon rank sum test was used. For multiple comparisons, a Kruskal–Wallis rank sum test was used.

## RESULTS

### Cohorts

Data on 219 children transplanted in Cohort 1 were available. We identified 117 patients who received a HSCT and had at least one CMV‐positive laboratory result, forming the ‘CMV infected’ dataset. Of these 117 patients, 68 received one or more anti‐CMV drugs, forming the Cohort 1 ‘CMV‐treated’ dataset. For Cohort 2, there were 342 HSCT recipients, of which 98 were in the ‘CMV‐infected’ dataset and 64 patients in the ‘CMV‐treated’ dataset. The demographics of the ‘CMV‐treated’ groups in Cohorts 1 and 2 are shown in Table 1.

**TABLE 1:** Demographics of ‘CMV‐treated’ patients in Cohort 1 and Cohort 2. Cohort 1: Paediatric haematopoietic transplant patients treated between 2010 and 2015; Cohort 2: Paediatric haematopoietic transplant patients treated between 2017 and 2021, post‐introduction of 1000 IU/mL pre‐emptive treatment threshold. ‘CMV‐treated’ patients had at least one positive CMV PCR laboratory test and received one or more anti‐CMV drugs.

Category	Cohort 1 (*n* = 68)	Cohort 2 (*n* = 64)
Age at transplant		
0 to <2	20 (29.4%)	12 (18.8%)
2 to <6	22 (32.4%)	17 (26.6%)
6 to <12	22 (32.4%)	24 (37.5%)
12 to <18	4 (5.9%)	11 (17.2%)
Sex		
Male	41 (60.3%)	36 (56.3%)
Female	27 (39.7%)	28 (43.8%)
Diagnostic category		
Malignant and benign haematology	29 (42.6%)	46 (68.6%)
Inherited immune deficiency	36 (52.9%)	16 (25.0%)
Metabolic disease	3 (4.4%)	2 (3.1%)
GvHD		
None	27 (39.7%)	18 (28.1%)
Grade I–II	24 (35.3%)	41 (64.1%)
Grade III–IV	9 (13.2%)	5 (7.8%)
NA	8 (11.8%)	0 (0%)
Conditioning		
Myeloablative	44 (64.7%)	39 (60.9%)
Non‐myeloablative	24 (35.3%)	21 (32.8%)
Serotherapy		
Yes	36 (52.9%)	53 (82.8%)
No	32 (47.1%)	12 (18.8%)
Transplant serostatus		
D−R−	10 (14.7%)	1 (1.6%)
D−R+	14 (20.6%)	12 (18.8%)
D+R−	9 (12.2%)	8 (12.5%)
D+R+	35 (51.5%)	43 (67.2%)
Death		
Yes	29 (42.6%)	16 (25.0%)
No	39 (57.4%)	48 (75.0%)

Abbreviation: CMV, cytomegalovirus; GvHD, graft versus host disease.

**FIGURE 1:**
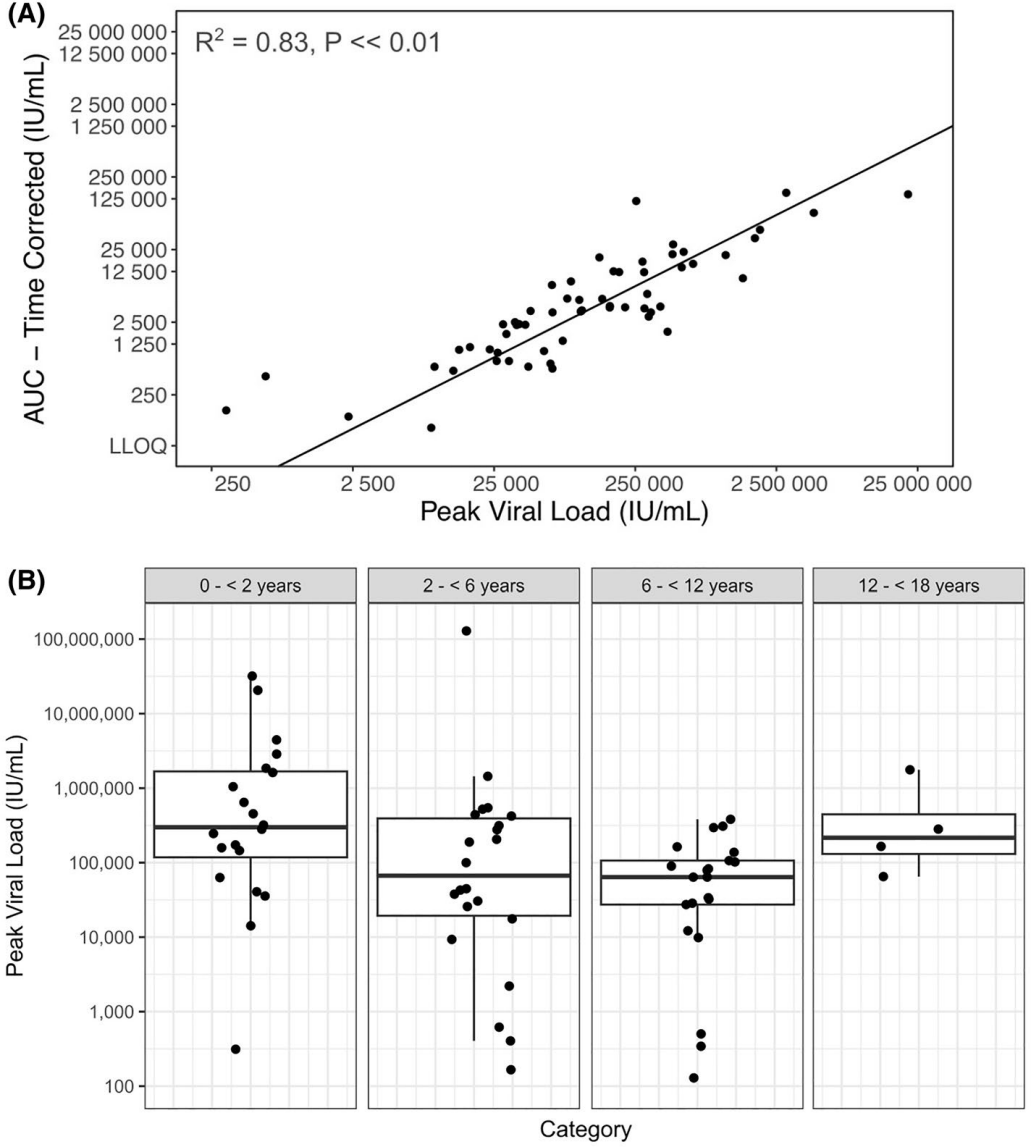
Peak CMV viral loads in CMV‐treated paediatric HSCT patients in Cohort 1 are strongly correlated with viral AUC and are not significantly related to age. (A) Time‐corrected CMV viral AUC correlates with peak viral load. Viral AUC values corrected for time and plotted against peak viral loads, with a log10‐linear model fitted to the data for the 68 CMV‐infected individuals in the ‘CMV treated’ dataset. (B) Peak viral loads for patients in the ‘CMV‐treated’ dataset. Peak viral loads of patients within the ‘CMV‐treated’ dataset (*n* = 68) split by age. Cohort 1: Paediatric haematopoietic transplant patients treated between 2010 and 2015 (pre‐introduction of 1000 IU/mL pre‐emptive treatment threshold). AUC, area under the curve; CMV, cytomegalovirus; HSCT, haematopoietic stem cell transplant.

To examine whether peak viral load is an appropriate representation of a patient's viral load profile, the AUC of the viral load measures for each of the 68 ‘CMV treated’ patients in Cohort 1 was calculated by trapezoid rule. This was then divided by the days constituting a patient's infection event to compute normalised, time‐corrected viral loads AUCs. Peak viral load was strongly correlated with the time‐corrected AUC (Figure 1A). A log_10_‐linear model was fitted to the pooled viral load statistics and was found to account for much of the variance in the data (*R* ^2^ = 0.83, *p* ≪ 0.01), with peak viral load appropriately representing much of the information contained within the time corrected AUC statistic.

We found that children <2 years of age exhibited the greatest peak CMV loads, although this trend did not reach statistical significance (Figure 1B). Unlike in adult transplant patients,^20^ we found viral load doubling time in children was highly variable both within and between individuals (range: 0.5–11.4 days) and therefore unreliable as a predictor of CMV severity. There was no correlation between peak viral load and viral doubling time (Pearson correlation coefficient −0.12).

### Model outputs for identification of optimal threshold

**FIGURE 2:**
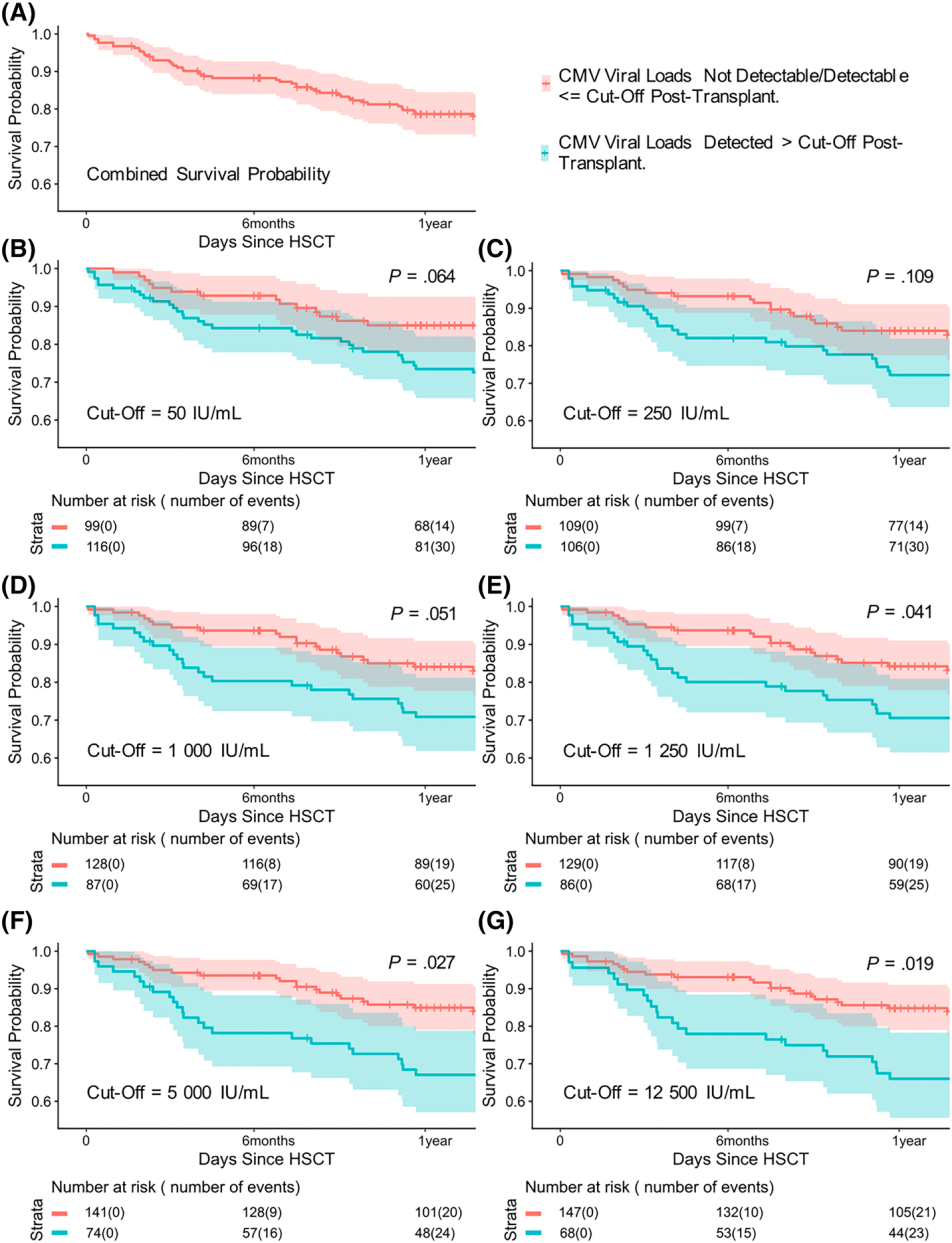
Peak CMV viral loads of 5000 IU/mL and above show clear separation in survival probability in Cohort 1. Cohort 1: Paediatric haematopoietic transplant patients treated between 2010 and 2015 (pre‐introduction of 1000 IU/mL pre‐emptive treatment threshold). CMV, cytomegalovirus.

Survival analyses were performed for patients in Cohort 1, testing six peak viral load thresholds (Figure 2). The thresholds used in the analysis started at the CMV lower limit of detection (50 IU/mL) and were incrementally raised to exceed 125 000 IU/mL.

CMV peak viral loads of 5000 IU/mL and above showed clear separation in survival probability, and a small further statistical difference existed between 5000 IU/mL and 250 IU/mL. The limited patient data between 1250 and 250 IU/mL mean that, although a separation in population mortality is suggested at lower thresholds, this was not statistically significant (Figure 2). A Mantel–Byar test was performed and confirmed significance for both 1250 IU/mL (*p* = 0.038) and 1000 IU/mL (*p* = 0.041).

Of the patients in our cohort whose peak viral load exceeded 1250 IU/mL, 78% had viral loads detected below this threshold prior to their viral load increase. This supported the suggestion that pre‐emptive treatment aimed at reducing the number of individuals whose viral load exceeds this value was an achievable aim in children undergoing HSCT. Given these findings, a treatment threshold of 1000 IU/mL was implemented on 1 January 2017.

### Reduction of peak viral, mortality and end‐organ disease following introduction of new treatment threshold

**FIGURE 3:**
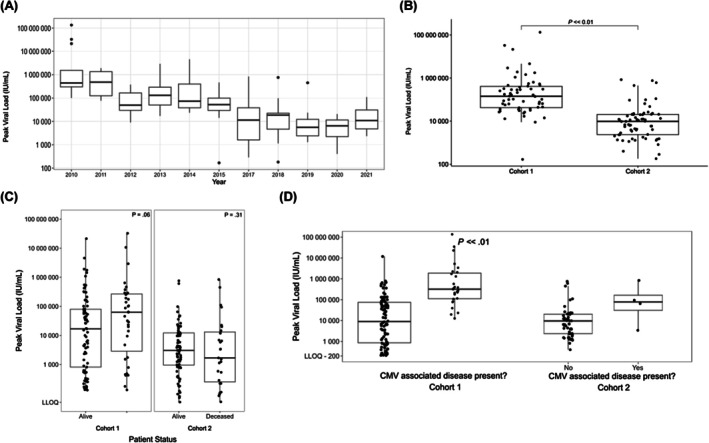
Peak CMV viral loads are significantly lower in Cohort 2 than in Cohort 1. (A) Trend of peak viral loads by year, with 2016 being the year of transition from 2500 IU/mL to 1000 IU/mL. Data from 2916 were excluded as there was mixed clinical practice that year around the new threshold introduction. (B) Peak viral loads in Cohort 1 and Cohort 2. Highly statistically significant reduction (*p* < 0.01) in Cohort 2 after new threshold introduction. (C) Peak viral loads in alive versus deceased patients in Cohort 1 and Cohort 2. No significant difference in peak viral loads between the two groups in either cohort. (D) Peak viral loads in Cohorts 1 and 2 analysed by the presence of CMV‐associated diseases. While there were highly statistically higher peak viral loads in patients with CMV‐associated disease in Cohort 1, the difference between patients with or without disease was not statistically different in Cohort 2. CMV, cytomegalovirus.

Following introduction of the treatment threshold, the peak CMV viral loads of paediatric HSCT recipients fell dramatically (Figure 3A,B). This was associated with a marked reduction in both mortality (36/117, 30.8% vs. 19/98, 19.4%) and the presence of end organ disease (Table 2). The reduction in the mortality rate was also associated with a loss of association between peak viral load and survival (Figure 3C) and CMV disease (Figure 3D), with all groups having a lower peak viral load than prior to the introduction of the treatment threshold. This may reflect that CMV disease was a smaller contributor to adverse outcomes following the introduction of the treatment threshold; while those with CMV disease still had higher peak viral loads than those without CMV disease, only four patients were considered to have had CMV disease in Cohort 2 (Figure 3D).

**TABLE 2:** Outcome for CMV‐infected individuals before and after the introduction of the treatment threshold. Patients with suspected CMV encephalitis, pneumonitis, retinitis and colitis in all individuals who were considered for transplant and exhibited >1 positive CMV laboratory result, making up the ‘CMV‐infected’ dataset. Cohort 1: Paediatric haematopoietic transplant patients treated between 2010 and 2015; Cohort 2: Paediatric haematopoietic transplant patients treated between 2017 and 2021, post‐introduction of 1000 IU/mL pre‐emptive treatment threshold.

Condition	Cohort 1 (*n* = 117)	Cohort 2 (*n* = 98)
CMV‐associated disease	27 (23.1%)	4 (4.1%)
CMV‐associated encephalitis	5 (4.3%)	1 (1.0%)
CMV‐associated pneumonitis	17 (14.5%)	3 (3.1%)
CMV‐associated retinitis	11 (9.4%)	2 (2.0%)
CMV‐associated colitis	2 (1.7%)	0 (0.0%)
Death	36 (30.8%)	19 (19.4%)

Abbreviation: CMV, cytomegalovirus.

## DISCUSSION

In Cohort 1, 53.9% (117/219) of HSCT patients developed CMV viraemia within 2 years post‐transplant. This is in keeping with incidence in previous studies, which vary widely from 30% to 66%.^3^, ^21^, ^22^ As has been observed for adult HCST and renal transplant patients, peak CMV viral loads in Cohort 1 correlated with the presence of end‐organ disease in the paediatric HSCT recipients. Although we classified disease in our patients with CMV as ‘None’, ‘Possible’, ‘Probable’ and ‘Proven’, we found no statistically significant difference between the peak viral loads for the last three, suggesting that they might be one entity. We suspect that, because of difficulties in obtaining microbiological or histological samples to confirm CMV end‐organ involvement in some patients in the ‘Possible’ and ‘Probable’ groups, the number of confirmed CMV disease cases was likely underestimated.

There is little literature to contextualise our findings on the mortality of CMV disease post‐HSCT in paediatric patients, which was similar in Cohort 1 to what has been observed in adults.^20^ Compared to adults, children are more likely to have primary rather than secondary CMV infection, placing them at higher risk of severe CMV infection and death. Furthermore, children undergoing HSCT are more likely to have a primary immunodeficiency than malignancy as the indication, increasing their propensity to develop CMV disease.^20^

Children also differ from adult transplant patients^23^, ^24^ in that their viral load doubling times are highly variable both within and between individuals. CMV viral loads frequently did not fit the exponential growth trajectory assumed between base and peak observations, and thus doubling time as a statistic was not representative of the growth kinetics observed in vivo. Individual host and treatment‐related effects provide further confounding to analyses dependent on doubling time. It is possible that these findings reflect the greater heterogeneity of underlying diagnoses at our centre compared to published adult cohorts. The unreliability of viral doubling time as a predictor of CMV severity in children places even more of an onus on implementing optimal thresholds for starting treatment.

We show a clear relationship between peak CMV viral load and survival, with the relationship holding over 1‐year post‐transplant. Individuals who reach peak CMV loads greater than 1250 IU/mL consistently had a significantly lower probability of survival. The overlapping confidence intervals in our time‐to‐event model around the first 100 days post‐HSCT indicate that, although viral load may influence mortality in the early post‐transplant period, the impact of CMV infections may be cumulative over time. While viral load thresholds for the treatment of CMV in large HSCT populations have previously been modelled in adults,^25^ this study is, to the best of our knowledge, the first time that such thresholds have been investigated specifically for children undergoing HSCT, representing an important milestone for the management of this group.

Given the clear relationship between peak viral loads of ≥1250 IU/mL and increased mortality, our unit chose to lower the threshold for starting pre‐emptive treatment from 2500 to 1000 IU/mL. This change was associated with a decrease in the average CMV viral load in our HSCT cohort and a statistically significant reduction in the proportion of patients with CMV disease (*p* < 0.01). Interestingly, the strong association between viral load and mortality in the CMV‐infected cohort, which had been present, was no longer observed after the introduction of the 1000 IU/mL threshold. This may reflect the overall reduction in the proportion of children dying, perhaps secondary to controlling CMV.

A limitation of this study is the difficulty in confirming causation with regard to viral load and mortality. Although higher CMV loads are associated with lower probability of survival, it is uncertain whether CMV viral load directly contributed to death, except in cases where the cause of death was specifically related to CMV end‐organ disease. Higher CMV viral loads may contribute to worse outcomes through indirect effects, such as fungal or bacterial infections, or renal dysfunction.^26^, ^27^ The study findings are limited to paediatric transplant recipients with CMV viral loads sampled from whole blood. It is not possible to extrapolate the findings from whole blood to plasma and previous studies have demonstrated only moderate concordance between the two.^28^ These data were also collected before the widespread use of letermovir as a CMV prophylactic agent in paediatric clinical practice.^7^ Repeating this analysis once sufficient data have been collected in the era of CMV prophylaxis post‐HSCT will be informative on its impact on CMV viral load dynamics and consequently on morbidity and mortality. Finally, another limitation to determining causality between the new CMV pre‐emptive treatment threshold and the reduced CMV‐related morbidity is the demographic differences between Cohort 1 and Cohort 2, with Cohort 1 having a higher proportion of children with inherited immune defects than Cohort 2 (52.9% vs. 25.0%). While the current model offers insight into the optimal timing for initiating pre‐emptive therapy, there remains a lack of data to inform when treatment can be safely discontinued. Although this question lies beyond the scope of the present study, it is an important avenue for future research, ideally supported by data specifically collected to inform such modelling.

Overall, these data strongly suggest that the 1000 IU/mL treatment threshold contributed to a reduction of CMV‐related adverse outcomes in post‐HSCT patients at our centre. Arguably, the threshold could have been reduced further, especially as there is always a delay between sampling and initiating treatment during which time CMV viral loads continue to rise. However, given the uncertainty around peak viral load thresholds below 1250 IU/mL due to low numbers, the lack of a relationship between CMV disease and doubling time, and the concerns around antiviral resistance, 1000 IU/mL as a threshold for starting treatment was felt to be a good starting point.

## AUTHOR CONTRIBUTIONS

A.D. performed clinical data analysis, prepared tables and wrote the manuscript. O.C. conceived the study, conducted data processing and analysis, generated figures and wrote the manuscript. B.K.M. contributed to data processing and analysis and participated in the initial study conception. J.B. contributed statistical expertise, supervision of O.C. and B.K.M. J.R.B., T.B., P.A., R.C., J.S., A.W. and J.F.S. provided clinical expertise, implemented clinical changes highlighted in the study and reviewed and commented on the manuscript. S.F. and K.R. provided clinical expertise and collected data. E.W. conceived the study, provided supervision and reviewed and commented on the manuscript. E.G. conducted data collection and analysis, contributed to study conception and manuscript writing and provided supervision. J.B. provided overall supervision, contributed to study conception and critically reviewed the manuscript.

## CONFLICT OF INTEREST STATEMENT

The authors declare no conflicts of interest.
